# Longitudinal stability in cigarette smokers of urinary biomarkers of exposure to the toxicants acrylonitrile and acrolein

**DOI:** 10.1371/journal.pone.0210104

**Published:** 2019-01-04

**Authors:** Menglan Chen, Steven G. Carmella, Chistopher Sipe, Joni Jensen, Xianghua Luo, Chap T. Le, Sharon E. Murphy, Neal L. Benowitz, F. Joseph McClernon, Ryan Vandrey, Sharon S. Allen, Rachel Denlinger-Apte, Paul M. Cinciripini, Andrew A. Strasser, Mustafa al’Absi, Jason D. Robinson, Eric C. Donny, Dorothy Hatsukami, Stephen S. Hecht

**Affiliations:** 1 Masonic Cancer Center, University of Minnesota, Minneapolis, Minnesota, United States of America; 2 Division of Biostatistics, School of Public Health, University of Minnesota, Minneapolis, Minnesota, United States of America; 3 Department of Medicine, University of California, San Francisco, California, United States of America; 4 Department of Psychiatry and Behavioral Sciences, Duke University, Durham, North Carolina, United States of America; 5 Department of Psychiatry and Behavioral Sciences, Johns Hopkins University, Baltimore, Maryland, United States of America; 6 Department of Family Medicine and Community Health, University of Minnesota Medical School, Minneapolis, Minnesota, United States of America; 7 Department of Behavioral and Social Sciences, Brown University, Providence, Rhode Island, United States of America; 8 Department of Behavioral Science, University of Texas MD Anderson Cancer Center, Houston, Texas, United States of America; 9 Department of Psychiatry, University of Pennsylvania, Philadelphia, Pennsylvania, United States of America; 10 Behavioral Medicine Laboratories, University of Minnesota Medical School, Duluth, Minnesota, United States of America; 11 Department of Physiology and Pharmacology, Wake Forest School of Medicine, Winston-Salem, North Carolina, United States of America; National Yang-Ming University, TAIWAN

## Abstract

The urinary metabolites cyanoethyl mercapturic acid (CEMA) and 3-hydroxypropyl mercapturic acid (3-HPMA) have been widely used as biomarkers of exposure to acrylonitrile and acrolein, respectively, but there are no published data on their consistency over time in the urine of cigarette smokers. We provided, free of charge over a 20 week period, Spectrum NRC600/601 research cigarettes to cigarette smokers in the control arm of a randomized clinical trial of the reduced nicotine cigarette. Urine samples were collected at weeks 4, 8, 12, 16, and 20 and analyzed for CEMA and 3-HPMA, and total nicotine equivalents (TNE) using validated methods. Creatinine-corrected intra-class correlation coefficients for CEMA, 3-HPMA, and TNE were 0.67, 0.46, and 0.68, respectively, indicating good longitudinal consistency for CEMA, while that of 3-HPMA was fair. A strong correlation between CEMA and TNE values was observed. These data support the use of CEMA as a reliable biomarker of tobacco smoke exposure. This is the first report of the longitudinal stability of the biomarkers of acrylonitrile and acrolein exposure in smokers. The data indicate that CEMA, the biomarker of acrylonitrile exposure, is consistent over time in cigarette smokers, supporting its use. While 3-HPMA levels were less stable over time, this biomarker is nevertheless a useful monitor of human acrolein exposure because of its specificity to this toxicant.

## Introduction

**Fig 1:**
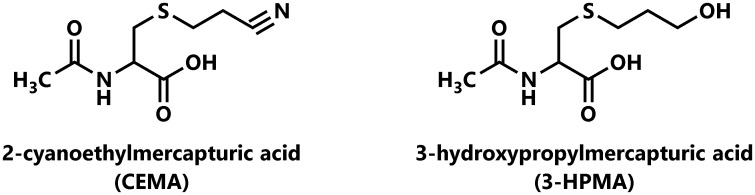
Structures of CEMA and 3-HPMA.

The U.S. National Toxicology Program 14^th^ Report on Carcinogens classifies acrylonitrile as “reasonably anticipated to be a human carcinogen” based on sufficient evidence of carcinogenicity from studies in experimental animals [1]. Acrylonitrile causes a variety of tumors in rats including cancer of the central nervous system and Zymbal gland. However, data from epidemiological studies are considered inadequate to evaluate specific exposures to acrylonitrile as causes of human cancer. The International Agency for Research on Cancer concluded that acrylonitrile is “possibly carcinogenic to humans.” [2] Tobacco smoke is a major source of human exposure to acrylonitrile, with a mean value of 28.4 μg per cigarette in mainstream smoke of U.S. brand cigarettes using the Health Canada smoking regimen [3]. Cyanoethyl mercapturic acid (CEMA, Fig 1) is a urinary metabolite of acrylonitrile formed by its initial Michael addition reaction with glutathione followed by metabolic removal of the glutamic acid and glycine residues by peptidases and *N*-acetylation of the remaining cysteine residue [4].

Jakubowski *et al* were the first to measure CEMA in humans exposed to acrylonitrile. They used gas chromatography with flame ionization detection [5]. Schettgen *et al* developed a column-switching liquid chromatography tandem mass spectrometry (LC-MS/MS) method for CEMA in human urine and found a relationship to cigarette smoking as well as passive smoke exposure [6]. Scherer *et al* reported a method for CEMA in human urine using derivatization with pentafluorobenzyl bromide followed by LC-MS/MS analysis. They observed a significant decrease in CEMA levels upon smoking cessation [7]. Minet *et al* showed that smokers excreted from 75–165 times more urinary CEMA than non-smokers and that CEMA levels in smokers were significantly correlated with total nicotine equivalents (TNE) and other biomarkers [8]. Multiple other investigators have confirmed and extended these results, including application to studies of marijuana smokers, waterpipe users, and e-cigarette users [9–21].

The second toxicant investigated in this study is acrolein, an intense eye and respiratory tract irritant which causes a variety of adverse effects including irritation, inflammation, and cell proliferation, although there is scant evidence for its carcinogenicity [22]. It is considered to be one of the most toxic compounds in tobacco smoke [23, 24]. Its respiratory toxicity results from its chemical reactivity with respiratory tract proteins and other macromolecules, depletion of glutathione, and necrotic cell death among other effects [23, 24]. Tobacco smoke is an important source of acrolein exposure, with a mean level of 177 μg per cigarette in mainstream smoke [3]. 3-Hydroxypropyl mercapturic acid (3-HPMA, Fig 1) is an accepted biomarker of acrolein exposure. This urinary metabolite forms by Michael addition of glutathione followed by endogenous reduction of the aldehyde group and metabolic processing to the *N*-acetylcysteine conjugate 3-HPMA.

Mascher *et al* were the first to develop an LC-MS/MS method for 3-HPMA, applying it to analysis of urine from smokers and non-smokers [25]. Scherer *et al* demonstrated a borderline significant decrease in the amount of 3-HPMA in the urine of smokers who used charcoal filtered cigarettes compared to cellulose acetate filtered cigarettes [26]. We demonstrated a significant decrease in urinary 3-HPMA when cigarette smokers stopped smoking [27, 28]. Yan *et al* developed a rapid LC-MS/MS method for analysis of 3-HPMA in urine [29]. Morin *et al* and Shepperd *et al* demonstrated significant correlations of mouth level exposure to acrolein in smokers and 3-HPMA in urine [30, 31]. Minet *et al* carried out an inter-laboratory comparison of 3-HPMA levels in urine and found good inter-laboratory reproducibility [32]. We developed a high throughput LC-MS/MS method for 3-HPMA in cigarette smokers’ urine [33]. and demonstrated significantly lower levels of this acrolein metabolite in the urine of e-cigarette users than in cigarette smokers [34]. Alwis *et al* determined 3-HPMA levels in the NHANES study, 2005–2006, and found five times higher levels among tobacco smokers than non-tobacco users [35]. Kassem *et al* found that hookah smoking was a significant source of acrolein exposure based on urinary 3-HPMA measurements [36]. We observed that urinary levels of 3-HPMA were significantly different among five ethnic groups of cigarette smokers in the Multiethnic Cohort Study [37].

Thus, multiple reports in the literature have described the development and application of CEMA and 3-HPMA as biomarkers of acrylonitrile and acrolein exposure, respectively, focusing mainly on exposure to these significant toxicants from cigarette smoking. However, to our knowledge, there have been no reports on the longitudinal consistency of these two important biomarkers of volatile toxicant uptake in cigarette smokers. It is critical to know whether measurement of a urinary biomarker at a given point in time is representative of its levels over time in cigarette smokers. We addressed this question here by examining the consistency over time of CEMA and 3-HPMA using urine samples obtained at 5 time points over 20 weeks from smokers participating in the control arm of a clinical trial of reduced nicotine content cigarettes [38]. These smokers were given, free of charge, “Spectrum” research cigarettes containing 15.5 mg nicotine per gram tobacco.

## Materials and methods

### Study design

The data reported here are from the control group of a 10-site randomized clinical trial that studied the effect of immediate vs. gradual reduction in nicotine content of cigarettes on smoking-related behavior and biomarkers of smoke exposure. Details of this multi-site trial have been reported [38]. The study protocol was approved by the University of Minnesota Institutional Review Board (IRB Code Number 1106M01561, FWA Number 00000312). Written consent was obtained from all subjects. Briefly, the control group consisted of 249 cigarette smokers (43% female) randomly assigned to smoke Spectrum NRC600/601 research cigarettes, which are non-menthol and mentholated, respectively, containing 15.5 mg nicotine/g tobacco, similar to commercially available cigarettes. The NRC600 cigarette mainstream smoke contained 54.9 μg/cigarette acrolein (ISO conditions) and 104 μg/cigarette acrolein under Canadian Intense conditions. The corresponding values for NRC601 cigarette smoke were 54.1 and 101 [39]. Data for acrylonitrile levels in mainstream smoke of these cigarettes was not available. The cigarettes were provided free of charge to participants over a period of 20 weeks. Participants attended a weekly clinic visit for the first 4 weeks and then bi-weekly visits for the next 16 weeks to complete questionnaires and other measures and receive supplies of additional cigarettes, approximately twice their self-reported baseline level of consumption. At all visits, smokers were advised on the importance of smoking only the study cigarettes, as long as they continued to smoke. Support was given for attempts to quit smoking if interest were expressed. At weeks 4, 8, 12, 16, and 20, first morning urine voids were collected for measurement of biomarkers.

### Analysis of CEMA and 3-HPMA in urine

The analysis was performed essentially as described [33] with slight modifications for analysis of CEMA. Briefly, [CD_3_]3-HPMA and [CD_3_]CEMA were added to 200 μl urine. An Oasis Max 60 mg mixed mode anion exchange 96 well plate (Waters Corp.) was preconditioned with methanol and 2% aqueous NH_4_OH. The sample was applied, and eluted with 30% methanol containing 2% formic acid, to give the fraction containing 3-HPMA and CEMA. The fraction was concentrated to dryness, and the residue dissolved in 60 μl MeOH/NH_4_OAc:1/3, pH 6.8, and analyzed by atmospheric pressure chemical ionization (APCI)-LC-MS/MS-SRM using an Agilent 1100 HPLC system (Agilent Technologies) coupled to a TSQ Quantum Discovery Max instrument (Thermo Scientific) equipped with a 50 x 3.0 mm, 2.5-micron Sunfire C18 LC column (Waters Corp.). Solvent A was 15 mM NH_4_OAc, pH 6.8, and solvent B was methanol. The column temperature was 50 °C and the flow rate was 0.4 ml/min. Three μL of the sample were injected. The LC solvent system was 98% A and 2% B held for 2 min, then ramped to 12% B in 6.5 min, then to 70% B in 2.5 min, and then to 2% B in 0.5 min and held for 6 min. The MS/MS system was run in the negative APCI mode using the following ion mass transitions for detection: 3-HPMA, *m/z* 220 → *m/z* 91; [CD_3_]3-HPMA, *m/z* 223 → *m/z* 91; CEMA, *m/z* 215 → *m/z* 162, [CD_3_]CEMA, *m/z* 218 → *m/z* 165. Other MS parameters were as follows: collision energy (V), 3-HPMA, 14; CEMA, 10; peak width, Q1 and Q3 (FWHM) 0.7; scan width (*m/z*), 0.40; scan time (sec), 0.2; vaporizer temp (°C), 450; capillary temperature (°C), 200; N_2_ sheath gas pressure, psi, 30; N_2_ auxiliary gas pressure, psi, 5.

### Method validation sample

Aliquots of urine from 9 cigarette smokers who smoked an average of 16 cigarettes per day were pooled together and spiked with various amounts of CEMA ranging from 50 to 600 ng/ml.

### Validation parameters

Intra-day precision was determined by repetitive analysis (N = 6) of the pooled smokers’ urine sample on a single day. Inter-day precision was determined by including the pooled smokers’ urine sample in each set of samples analyzed during the study (N = 129). Accuracy was determined by adding known amounts of CEMA to a pooled smokers’ urine sample and then carrying out the analysis.

### Analysis of total nicotine equivalents (TNE) and creatinine in urine

TNE (sum of nicotine, cotinine, 3’-hydroxycotinine and their glucuronides and nicotine-*N*-oxide) and creatinine were quantified as described previously [40].

### Statistical analyses

Summary statistics, including mean, median, and interquartile ranges, for creatinine-corrected 3-HPMA, CEMA, and TNE at weeks 4, 8, 12, 16, and 20 are presented using boxplots. The intraclass correlation coefficient (ICC) and 95% confidence intervals (CI) were estimated by fitting a linear mixed model for repeatedly measured, log-transformed biomarkers with a random intercept using the SAS macro%ICC9 [41] where missing visits were assumed to be at random. The coefficient of variation (CV) for each participant who had two or more measurements was calculated as the sample standard deviation (SD) divided by the sample mean; and then the mean CV was calculated for all such participants. Note that higher ICC and lower CV correspond to better longitudinal stability. The repeated-measure correlation (*r*_*rm*_) between two log-transformed biomarkers and 95% CI were estimated by using the R package rmcorr; [42] the scatterplot of raw data was presented together with parallel lines which were fitted to the data of each subject. Note that the repeated-measure correlation is invariant to creatinine correction (or any other biomarker corrections or no correction) because of the log-transformation.

## Results

The study sample consisted of 249 smokers (43% female), mean age 45.0 ± 13.4 years. They were 61% Caucasian, 30% African American, and 9% other races. They smoked a mean of 20.6 ± 11.4 cigarettes per day during the 20 week study period; 97.7% of these were the Spectrum research cigarettes, based on self-report. Further characteristics of the group have been reported [38].

**Fig 2:**
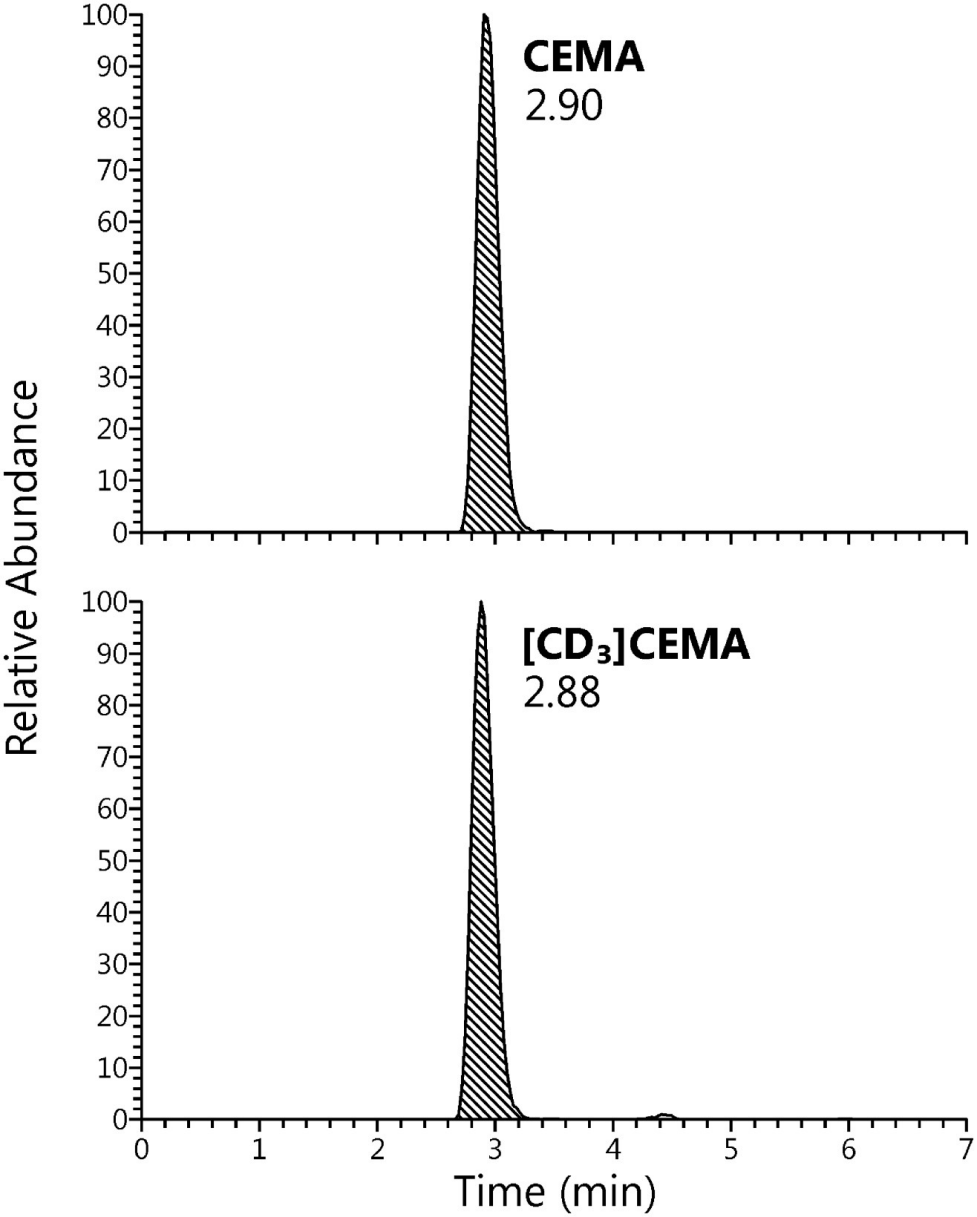
LC-MS/MS chromatograms of CEMA and the internal standard [CD_3_]CEMA.

**Fig 3:**
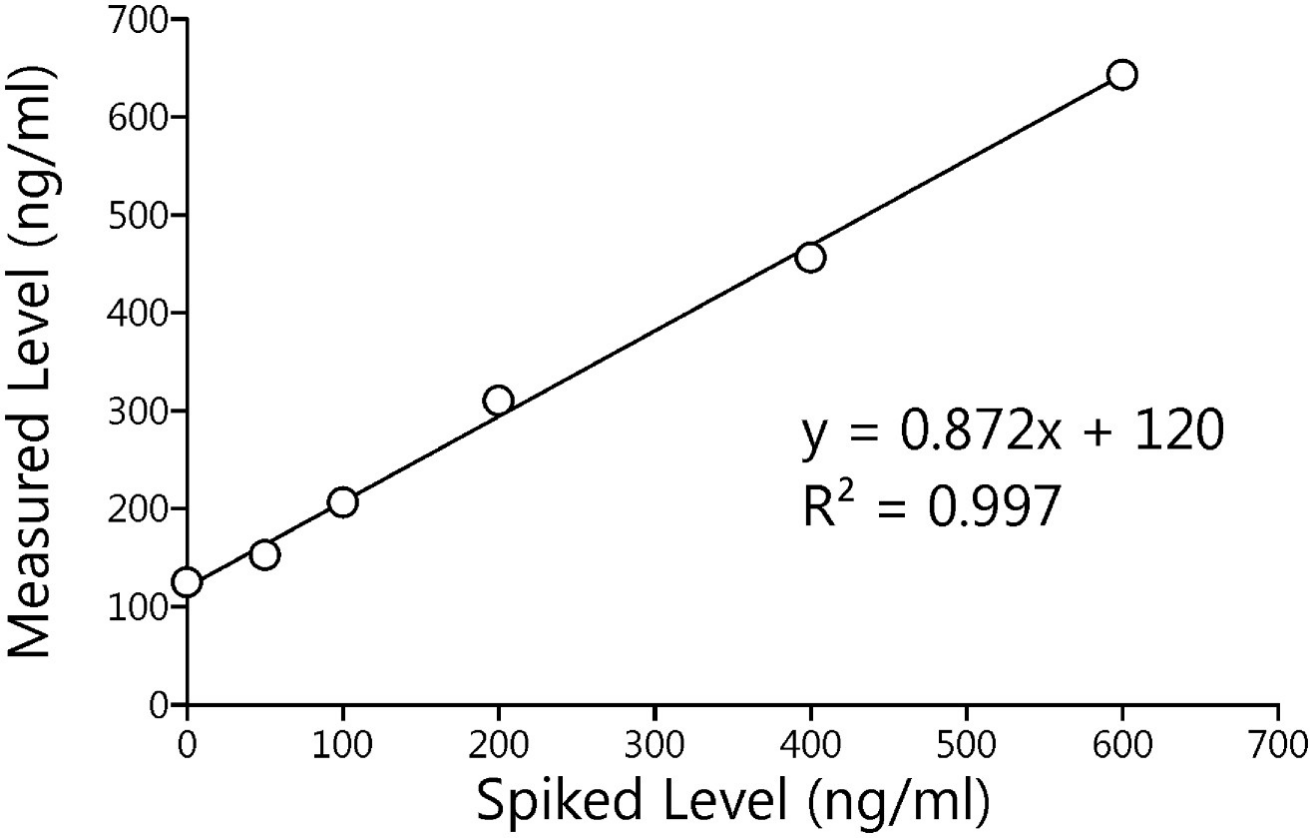
Relationship between added and measured CEMA in urine. MW of CEMA = 216.

The analytical method has been previously validated for 3-HPMA [33]. In this study, validation of the method was extended to CEMA. An LC-MS/MS chromatogram of CEMA and the internal standard [CD_3_]CEMA is illustrated in Fig 2. As shown in Fig 3, there was excellent agreement between amounts of CEMA quantified in a pooled smokers’ urine sample, and the amounts added to that sample (R^2^ = 0.997). The expected value for CEMA in the unspiked urine sample was 117 ± 7.50 ng/ml (S.D.) and the observed y-intercept value was 120 ng/ml (0.56 nmol/ml). Intra-day precision (N = 6, CV x 100%) was 6.40% and inter-day precision (N = 129) was 6.60%. The limit of quantitation was 0.18 pmol/ml.

**Fig 4:**
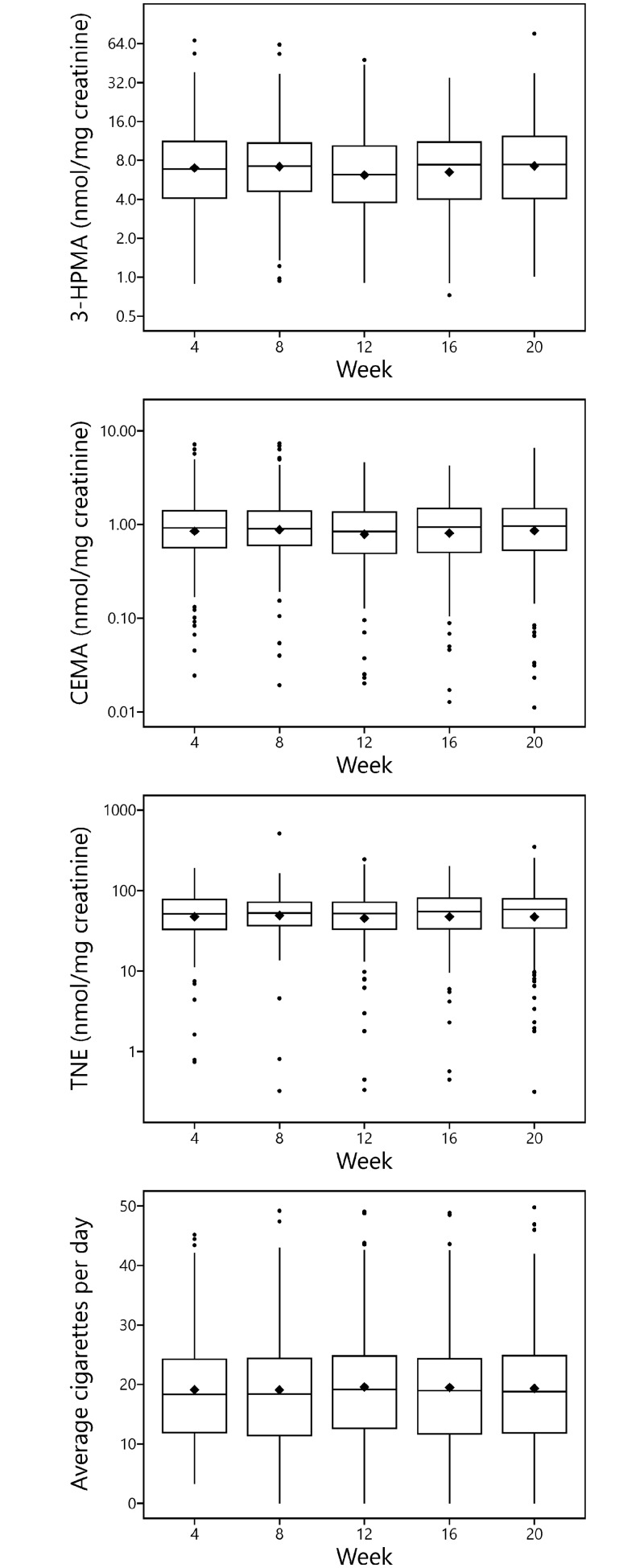
Mean values of 3-HPMA, CEMA, TNE, and CPD over the 20 week period in which subjects smoked the Spectrum cigarettes. Horizontal line inside the box: median; black diamond: mean; bottom and top edge of the box: 1^st^ and 3^rd^ quartile (interquartile range [IQR]); the upper whisker extends from the top of the box to the largest value no further than 1.5 times IQR and the bottom whisker extends from the bottom of the box to the smallest value no further than 1.5 times IQR; the y-axis is in natural log scale.

**Fig 5:**
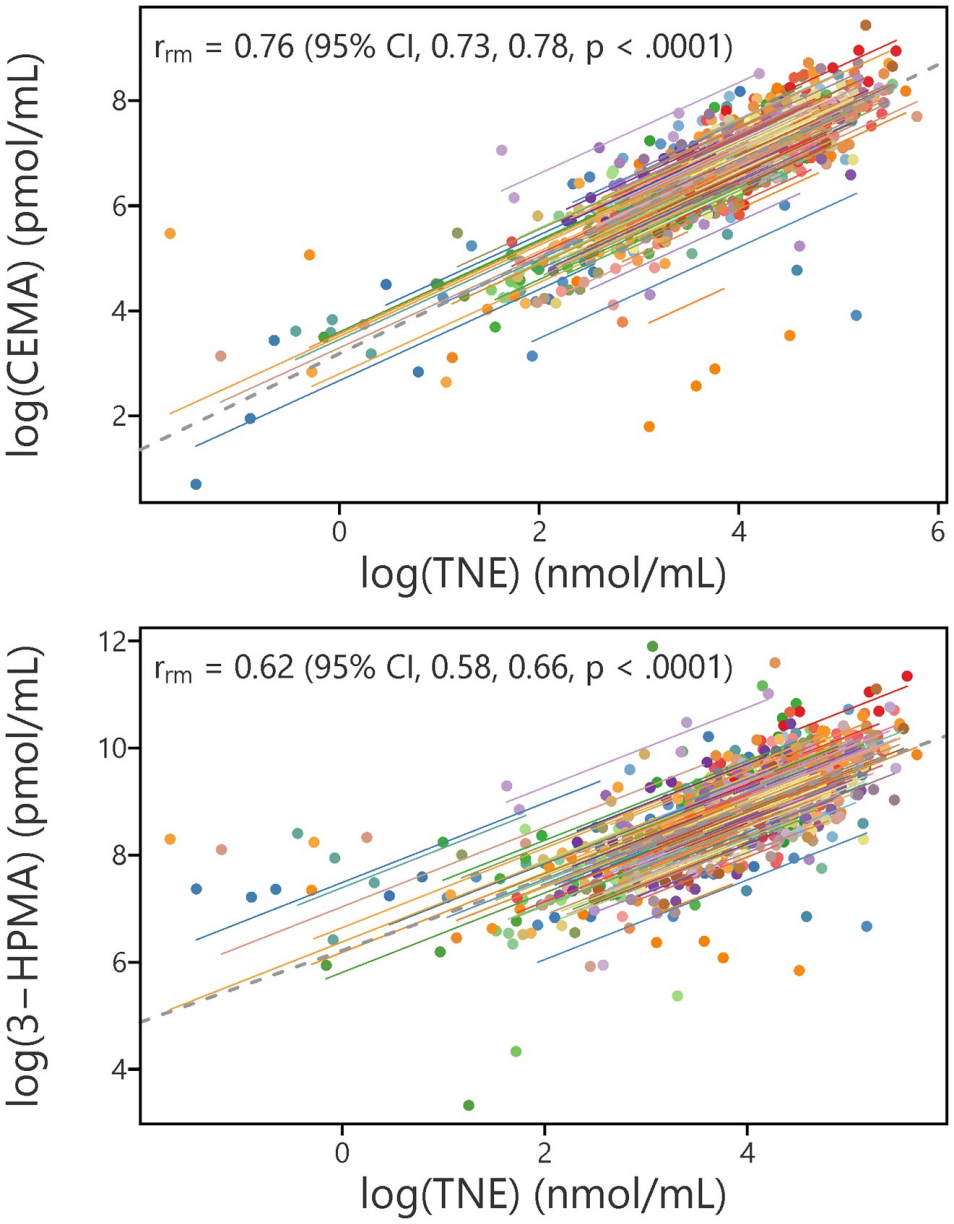
Correlation of CEMA and 3-HPMA with TNE; each individual is represented by a different color.

Means and other summary statistics for 3-HPMA, CEMA, and TNE over the course of the 20 week period are shown in Fig 4. Both 3-HPMA and CEMA were significantly correlated with TNE; the correlation of log transformed CEMA with TNE (*r*_*rm*_^2^ = 0.58) was stronger than that of 3-HPMA (*r*_*rm*_^2^ = 0.38) (Fig 5).

For participants who provided 2 or more urine samples (N = 224), the mean CV for creatinine corrected 3-HPMA, CEMA, and TNE were 48%, 41%, and 33%, respectively. The ICC, expressed per mg creatinine and per ml urine (e.g., non-corrected), are summarized in Table 1. For the creatinine-corrected data, the ICC of CEMA was similar to that of TNE and significantly superior to that of 3-HPMA. The ICC of the non-corrected values shared the same pattern as that of the creatinine-corrected values (see Table 1).

**Table 1:** Estimated intra-class correlation coefficients (ICC) for 3-HPMA, CEMA, and TNE.

Variable	N	Creatinine-corrected ICC (95% CI)	Non-corrected ICC (95% CI)
3-HPMA	233	0.46 (0.39, 0.52)	0.38 (0.32, 0.45)
CEMA	234	0.67 (0.62, 0.72)	0.55 (0.49, 0.61)
TNE	236	0.68 (0.63, 0.72)	0.58 (0.52, 0.64)

Creatinine-corrected values were calculated using nmol/mg creatinine. Non-corrected values were calculated using nmol/ml urine.

## Discussion

Our data demonstrate that the longitudinal consistency of CEMA (ICC, 0.67) is similar to that of TNE (ICC, 0.68) and superior to that of 3-HPMA (ICC, 0.46). Quantitation of CEMA and 3-HPMA as urinary biomarkers of exposure to acrylonitrile and acrolein, respectively, has been reported extensively, but to our knowledge this is the first study to investigate the longitudinal consistency of these biomarkers in humans. Longitudinal consistency is an important parameter as it provides an indication of the generalizability of a measurement taken at a particular point in time. The control group in a randomized clinical trial of reduced nicotine content cigarettes, as reported here, provided an ideal opportunity to test biomarker longitudinal consistency. This control group of daily cigarette smokers was provided with Spectrum NRC600/601 research cigarettes, free of charge, over a period of 20 weeks. They were asked to smoke only these cigarettes, thus providing a relatively stable source of smoke exposure to acrylonitrile and acrolein. According to Cicchetti [43], an ICC between 0.75 and 1.00 is “excellent,” one between 0.64 and 0.74 is “good”, while a value between 0.40 and 0.59 is “fair.” The similar ICC values of CEMA and TNE are clearly related to their relatively strong correlation, as indicated in Fig 5, which has also been noted by others [6, 8, 10, 14, 15]. We have previously determined ICC values for total urinary cotinine and total urinary 4-(methylnitrosamino)-1-(3-pyridyl)-1-butanol (NNAL) of 0.58 and 0.76, respectively, in smokers over a period of one year [44].

The better longitudinal consistency of CEMA as opposed to 3-HPMA undoubtedly results from the fact that exposure to acrolein from sources other than cigarette smoke is more likely than it is for acrylonitrile. Published studies [7, 8, 10–12] and our own unpublished data indicate that the relative levels of CEMA in smokers vs. non-smokers are far greater than those of 3-HPMA. In one recently completed unpublished study we observed a mean value of 0.31 ng/ml CEMA in the urine of 328 non-smokers analyzed by the method reported here, while the mean value in the current study was 300 ng/ml (1.38 nmol/ml), about 1000 times greater than in non-smokers. Others reported differences of 10–100 fold in CEMA values between smokers and non-smokers [6, 8, 10, 11, 15]. In contrast, data for 3-HPMA in our studies noted above were 443 ng/ml in the 328 non-smokers and 2190 ng/ml (9.9 nmol/ml) in smokers, a 6.7-fold difference, which is generally consistent with the literature [45]. These contrasting differences undoubtedly result from the fact that human environmental and endogenous exposure to acrylonitrile from sources other than tobacco smoke is relatively rare while acrolein has multiple sources, most notably endogenous formation resulting from lipid peroxidation, polyamine metabolism and related processes, in addition to multiple exogenous combustion processes [46]. The formation of acrylonitrile from tobacco nitrate and nitrite has been postulated based on pyrolysis experiments [47]. Other potential sources of acrylonitrile in cigarette smoke may include pyrolysis of nicotine and anatabine.

The use of first morning void urine samples in this study may also have contributed to the relatively poor longitudinal stability of 3-HPMA. A study by Sarkar *et al* [48] evaluated spot urine samples at three time points (early morning, post-lunch and evening) along with 24 h urine collections in cigarette smokers. They observed diurnal variation in 3-HPMA levels, with lower levels in morning spot urine collections than in post lunch or evening collections, in contrast to tobacco-specific biomarkers such as TNE.

The method reported here for quantitation of CEMA uses APCI as the MS ionization technique. Most, if not all, other methods for CEMA analysis report the use of electrospray ionization techniques. Our choice of APCI was based to some extent on our analytical method for 3-HPMA which followed descriptions of APCI techniques used for other mercapturic acids [27, 49]. Furthermore, some studies indicate that APCI is less susceptible to ion suppression than electrospray ionization [50]. Our data for CEMA in smokers are similar to those of other reports that used electrospray ionization and the validation parameters are also similar [6–8, 10, 11, 15]. Thus, both ionization techniques appear to be acceptable for CEMA quantitation, when coupled with appropriate LC conditions and MS instrument parameters.

In summary, we report data which strongly support the use of CEMA as a biomarker of acrylonitrile exposure in cigarette smokers. Consistent with published studies, we find that LC-MS analysis of CEMA is accurate, precise, and dependable, and that the data from smokers correlate with TNE. Unique to this study, we also demonstrate good longitudinal consistency of urinary CEMA in cigarette smokers sample over a 20 week period. While the longitudinal stability of 3-HPMA was lower than that of CEMA, it remains a valid and specific quantitative biomarker of acrolein exposure in humans.

## Supporting information

S1 Table Biomarker values. (XLS)
